# Contributions of Bioactive Molecules in Stem Cell-Based Periodontal Regeneration

**DOI:** 10.3390/ijms19041016

**Published:** 2018-03-28

**Authors:** An-Qi Liu, Cheng-Hu Hu, Fang Jin, Li-Shu Zhang, Kun Xuan

**Affiliations:** 1 State Key Laboratory of Military Stomatology & National Clinical Research Center for Oral Diseases & Shaanxi Clinical Research Center for Oral Diseases, Department of Pediatric Dentistry, School of Stomatology, The Fourth Military Medical University, Xi’an 710032, China; liuanqi@fmmu.edu.cn; 2 Research and Development Center for Tissue Engineering, The Fourth Military Medical University, Xi’an 710032, China; chenghu@xiterm.com (C.-H.H.); jinfang@fmmu.edu.cn (F.J.); lishu101@fmmu.edu.cn (L.-S.Z.); 3 State Key Laboratory of Military Stomatology & National Clinical Research Center for Oral Diseases & Shaanxi Clinical Research Center for Oral Diseases, Department of Orthodontics, School of Stomatology, The Fourth Military Medical University, Xi’an 710032, China

**Keywords:** bioactive molecules, periodontal regeneration, mesenchymal stem cells, cell aggregates/cell sheets, periodontal ligament stem cells

## Abstract

Periodontal disease is a widespread disease, which without proper treatment, may lead to tooth loss in adults. Because stem cells from the inflammatory microenvironment created by periodontal disease exhibit impaired regeneration potential even under favorable conditions, it is difficult to obtain satisfactory therapeutic outcomes using traditional treatments, which only focus on the control of inflammation. Therefore, a new stem cell-based therapy known as cell aggregates/cell sheets technology has emerged. This approach provides sufficient numbers of stem cells with high viability for treating the defective site and offers new hope in the field of periodontal regeneration. However, it is not sufficient for regenerating periodontal tissues by delivering cell aggregates/cell sheets to the impaired microenvironment in order to suppress the function of resident cells. In the present review, we summarize some promising bioactive molecules that act as cellular signals, which recreate a favorable microenvironment for tissue regeneration, recruit endogenous cells into the defective site and enhance the viability of exogenous cells.

## 1. Introduction

Periodontal disease represents a type of infectious disease that affects the tooth-adjacent supporting tissues, including the gingiva, periodontal ligament and alveolar bone. Based on its ability to cause alveolar bone loss, periodontal disease can be divided into gingivitis and periodontitis [1]. Gingivitis is the mildest form of periodontal disease with the highest prevalence, which is mainly caused by local bacterial biofilms attached to the surface of the tooth and gingiva. The gingiva represents the soft tissue attached to both the alveolar bone and the enamel surface. More importantly, it contains junctional epithelium, which tightly connects the tooth surface and the gingiva, thus protecting the periodontal ligament and alveolar bone from infection [2]. Due to the protective effects of the junctional epithelium, mild bacterial infections only result in gingivitis, which can be readily reversed by supragingival scaling and effective oral hygiene. However, periodontitis, especially severe periodontitis that is caused by local bacterial biofilms and also related to metabolic, genetic and immune diseases, results in lesions that lead to gingival bleeding, periodontal attachment loss and alveolar bone absorption [3].

Periodontitis is triggered by the interaction between the local bacterial biofilm and the host immune response against these bacteria [4]. When the bacteria invade the periodontal ligament (a special soft connective tissue that is rich in vessels and cells) that surrounds the roots of the tooth and connects the root cementum and alveolar bone to collagen fibers, both the host immune response and the bacteria release proteolytic enzymes and cytokines that damage the periodontal ligament. This results in an inflammatory microenvironment, which causes the loss of the periodontal ligament and the movement of the junctional epithelium towards the root. Because the height from the top of the junctional epithelium to the alveolar bone crest (known as the biologic width) is fixed, the loss of the periodontal ligament immediately initiates the absorption of the alveolar bone, which may finally lead to tooth loss [5]. Unfortunately, the absorption of the alveolar bone is irreversible by clinical routine methods, such as subgingival scaling and guided tissue regeneration, as these are mainly aimed at removing inflammation without or with little periodontal regeneration [6]. Therefore, the development of novel approaches for facilitating periodontal regeneration, including the reattachment of the periodontal tissues, regeneration of the periodontal ligament with same morphology and full function, along with a height increase of the alveolar bone, represents an urgent need and has become a target of extensive research.

With the development of stem cell therapies, it appears possible to achieve the complete restoration of lost periodontal tissues, including the periodontal ligament and the alveolar bone. Stem cell therapies have been developed for 50 years and have been proven effective in the field of hematopoietic diseases, such as lymphoma and leukemia [7]. Recently, mesenchymal stem cells (MSCs) have garnered considerable public attention as cell sources for tissue regeneration due to their potential to regenerate injured or pathologically damaged tissues to a normal and healthy state [8]. Moreover, MSCs isolated from the periodontal ligament (PDLSCs) tend to regenerate their tissue of origin, making them the best candidate for periodontal regeneration [9]. To obtain specific shapes that match the lost tissues and prevent collapse, various scaffolds have recently been engineered in an effort to enhance the effect of tissue regeneration [10,11,12,13].

We expect these scaffolds to fulfill several roles, including inducing the transplanted MSCs to migrate and differentiate into specific tissues, mobilizing endogenous MSCs to the damaged site and releasing certain growth factors persistently to recreate a favorable regeneration microenvironment [14,15]. To satisfy these requirements, some autologous grafting materials, such as cell aggregates/cell sheets [16], have been devised to reduce the drawbacks of presently existing scaffolds, such as immunological rejection; complicated techniques with multiple processes; and expensive cost.

However, merely applying autologous grafting materials is not enough to regenerate large periodontal defects because they not only lack a stable shape, but also lack sufficient cell signaling to induce the differentiation of MSCs into periodontal tissues [17].Consequently, bioactive molecules have been receiving increasing attention as promising agents for periodontal disease. These bioactive molecules are usually small molecules that possess both anti-inflammatory [18] and anti-oxidative [19] properties, alleviating the local inflammatory microenvironment and facilitating the further regeneration of the alveolar bone and soft tissues [20].

This review briefly introduces three types of novel bioactive molecules that have been investigated for periodontal regeneration. The majority of these studies have focused on the contribution of bioactive molecules as molecular signals in periodontal regeneration and on their function in recreating favorable microenvironment and enhancing the regeneration capacity of cell aggregates/cell sheets.

## 2. The Vital Role of Bioactive Molecules in Stem Cell-Based Periodontal Regeneration

**Figure 1:**
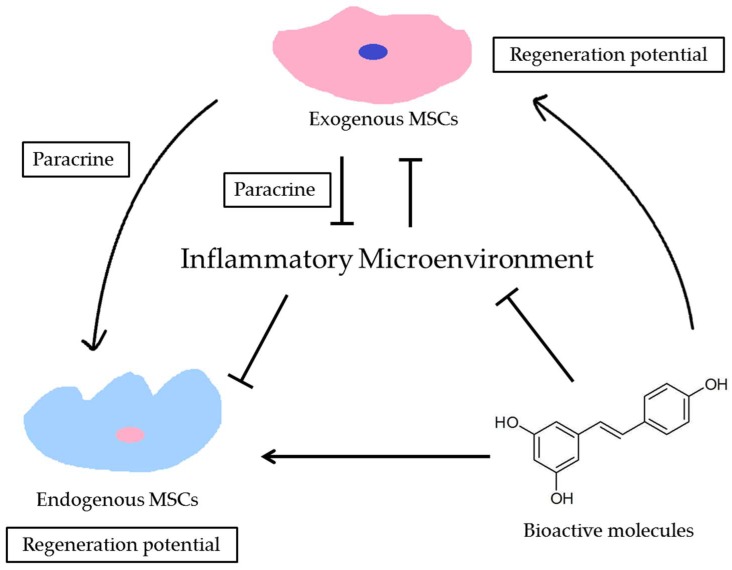
The schematic diagram of the interaction among bioactive molecules, mesenchymal stem cells (MSCs) and inflammatory microenvironment. Since inflammatory microenvironment could suppress the regeneration potential of both endogenous and exogenous MSCs, bioactive molecules could directly enhance regeneration potential of MSCs and recreate a favorable microenvironment for periodontal regeneration (Arrows represent positive effect and T-bars represent negative effect).

During the process of regeneration via tissue engineering, MSCs represent an indispensable factor. These cells proliferate and differentiate into various types of cells, before migrating in an orderly way to regenerate the defective tissue [8]. However, even MSCs isolated from the same tissue but from a different microenvironment can act differently [21]. Consequently, the status of the recipient microenvironment and its impact on the resident MSCs via a complex signal transduction network has gradually become apparent. Thus, rescuing the impaired microenvironment and promoting the regenerative potential of both endogenous and exogenous MSCs represent urgent issues that need to be addressed. Therefore, bioactive molecules have garnered attention as groups of biological signaling molecules that could not only replenish the signaling network responsible for the crosstalk in the impaired microenvironment, but also promote the MSCs-based periodontal regeneration [22] (Figure 1).

### 2.1. The Extracellular Microenvironment Releases Molecular Signals to Modulate MSCs

In healthy periodontal tissues, MSCs reside within a favorable microenvironment, which benefits stem cell self-renewal and maintains tissue homeostasis. However, when periodontitis or another periodontal injury occurs, this favorable microenvironment undergoes numerous sudden changes involving different cell types and soluble signals. The changes in the microenvironment also alter the function of resident MSCs. For instance, PDLSCs isolated from the periodontal microenvironment, which are known as inflamed periodontal ligament stem cells (I-PDLSCs), have a greater proliferation capacity but lower osteogenic, adipogenic and immunomodulatory potential than those in healthy PDLSCs (H-PDLSCs) [23]. In addition, I-PDLSCs display a high proliferative and migratory capacity but decreased osteogenesis when compared with H-PDLSCs derived from the same patient [21]. Moreover, in vivo studies have shown that I-PDLSCs have a lower bone regeneration capability after subcutaneous transplantation into the dorsa of nude mice [21].

As the function of MSCs is impaired in an inflammatory microenvironment, changing the adverse microenvironment into a favorable one is beneficial for the rejuvenation of the impaired MSCs. Our team has established a co-culture system of dental follicle cells (DFCs), which could provide a favorable microenvironment, and I-PDLSCs to investigate whether a beneficial microenvironment could modulate the activities of MSCs. Our results have demonstrated that DFCs can recreate a beneficial microenvironment for I-PDLSCs, which subsequently enhanced their proliferation and differentiation potential via cell-to-cell interactions or by secreting cellular factors as molecular signals [24]. Moreover, when aged PDLSCs were exposed to a young microenvironment supplied by the conditioned medium (CM) from young PDLSCs, the aged PDLSCs exhibited improved proliferation and differentiation. Furthermore, 8 weeks after the implantation of aged PDLSCs induced in a young microenvironment into the subcutaneous space of immunodeficient mice, these cells generated more mineralized tissues than those in the untreated aged groups [25].

These results indicate that changing the microenvironment where the MSCs reside could change their phenotypes and hence, lead to a better regeneration outcome [26]. It has been demonstrated that exogenous MSCs could regulate the behavior of endogenous MSCs through paracrine factors, which play a major role in the cell-to-cell interactions that facilitate tissue regeneration [27]. The administration of bioactive molecules could not only effectively alleviate the inflammatory microenvironment induced by periodontitis, but also act as the signal molecules binding to the specific cell membrane receptors of target cells to regulate cellular events in these cells.

### 2.2. MSCs Release Molecular Signals to Modulate the Microenvironment

Due to the unique regenerative potential of MSCs, they have become one of the most investigated cell types in cell-based therapies. By releasing anti-inflammatory cytokines to counteract the inflammatory microenvironment, expressing growth factors to facilitate the migration of endogenous MSCs to the impaired site and secreting immuno-modulatory proteins to change the host immune response, transplanted MSCs can change the microenvironment of injured tissues into a beneficial one to ensure MSCs-based regeneration [28]. Most recently, some cell-free therapies involving the usage of MSCs-derived CM or MSCs-derived extracellular vesicles (EVs), including exosomes, microvesicles and large oncosomes, have emerged. These therapies have attracted attention because they are hypoallergenic and highly targeted [29]. One study has reported that pre-treatment with PDLSCs-CM significantly increased the secretion of IL-10 anti-inflammatory cytokines in the motoneurons of lipopolysaccharide-exposed mice and enhanced neuronal growth [30]. This indicates that PDLSCs-secreted molecules possess anti-inflammatory and tissue regeneration capacities.

In general, MSCs could mediate the ambient microenvironment via cell-to-cell interactions, such as autocrine, cell contact and paracrine interactions. Paracrine signaling products include cytokines and EVs, which contain various mRNAs, microRNAs and proteins. These products can interact with receptors on the target cell membranes and sequentially activate the downstream signaling pathways. This results in specific cellular behaviors, such as cell migration and differentiation [27,31]. Thus, transplanted MSCs modulate the concentration of soluble factors and the types and density of host cells, which helps in recreating a favorable microenvironment for tissue regeneration.

### 2.3. Bioactive Molecules Act as Molecular Signals That Modulate MSCs and Recreate the Microenvironment

Over the years, numerous animal experiments and clinical trials have been performed in the field of periodontal regeneration. However, the results are still inconclusive and many issues remain to be addressed, including the best scaffold, the most suitable cell type, the specific indications and the start time for regenerative therapy [6]. Recently, by exploring the interactions between the microenvironment and the resident stem cells, we have gradually gained a better understanding of the mixed outcomes of periodontal regeneration studies. As the inflammatory microenvironment of periodontal impairment sites could suppress the regenerative potential of resident stem cells, it could also influence the regenerative potential of transplanted MSCs and lead to the failure of periodontal regeneration [22]. In this regard, the application of bioactive molecules could supplement molecular signals and effectively recreate a favorable microenvironment for regeneration. Furthermore, these bioactive molecules could themselves act as molecular signals, recruiting endogenous MSCs into the impaired sites, inducing exogenous MSCs to differentiate into specific tissues and enhancing cell-to-cell communication to promote periodontal regeneration.

## 3. Bioactive Molecules Associated with Periodontal Regeneration

Surgical and anti-inflammatory approaches, or a combination of these, represent conventional modes for the treatment of periodontitis. However, while these approaches are effective for controlling periodontitis, they are not sufficient for achieving adequate periodontal regeneration, which is defined by the reattachment of the periodontal ligament and the regeneration of the alveolar bone [32,33]. As described above, bioactive molecules could act as a bridge, bonding exogenous and endogenous MSCs in the impaired microenvironment and inducing them to differentiate into the specific tissues that need to be regenerated. Therefore, various bioactive molecules have been extensively explored to enhance the outcomes of periodontal regeneration. We further describe these in detail below (Table 1).

### 3.1. Growth Factors

Growth factors (GFs) are natural biological mediators that have long been thought to possess the potential to regulate key cellular events by binding to receptors on the target cell membranes and inducing intracellular signaling pathways [51]. Given that conventional treatment procedures cause dissatisfaction in both the clinician and the patient due to their suboptimal regenerative outcomes, GFs were suggested as a solution in order to enhance periodontal regeneration by alleviating the inflammatory microenvironment and increasing the proliferation and differentiation capacity of transplanted stem cells [37,43].

Through a variety of preclinical animal experiments and clinical trials, the US Food and Drug Administration (FDA) has approved an enamel matrix derivative (EMD) and the recombinant human platelet-derived growth factor-BB (rhPDGF-BB) as pharmaceuticals for the treatment of patients with periodontal defects [51]. Moreover, platelet concentrates (PCs), such as platelet-rich plasma (PRP) and platelet-rich fibrin (PRF) that serve as natural polymeric scaffolds rich in growth factors, are also currently widely applied in the field of periodontal regeneration.

EMD is derived from purified enamel matrix proteins that have shown favorable preclinical and clinical results [39,40]. Over the past 20 years, EMDs have been shown to facilitate osteoblast and PDLSCs adhesion, proliferation and differentiation. Hence, these factors are able to promote periodontal regeneration [34,35]. However, the real obstacle that limits its large-scale clinical application is the fluidity of its carrier gel. Without sufficient mechanical strength, it is difficult for EMD to prevent flap collapse and exert long-term effects, especially for large periodontal defects. Due to its fluid consistency, it requires the combination of various types of grafting materials to achieve satisfactory clinical outcomes [36]. Recently, a new carrier system for EMD has been designed by mixing EMD with bone grafts or collagen matrices. This has resulted in a significantly higher adsorption of amelogenin, the major ingredient of EMD, on absorbable collagen sponges when compared to previous methods in addition to enhanced periodontal regeneration [38]. Moreover, the precoating of EMD onto bone grafting particles significantly increased the adhesion, proliferation and differentiation potential of osteoblasts [38].

Another extensively characterized FDA-approved growth factor for periodontal regeneration is rhPDGF-BB. PDGF-BB is an isoform of PDGF that has been shown to facilitate periodontal regeneration after local administration [43,51]. Due to the short half-life of PDGF-BB, the use of recombinant PDGF-BB has been investigated for gene therapy. Clinical studies have demonstrated that a combination of purified recombinant human PDGF (rhPDGF) with synthetic ceramic matrices or a combination of rhPDGF-BB with bone allografts can both be used as periodontal regenerative agents. In addition, rhPDGF-BB in combination with freeze-dried bone allografts produced robust periodontal regeneration and improved the gingival attachment in interproximal bone defects and class II furcation defects. Therefore, this combination provides an effective treatment for severe periodontal bone loss [43]. However, the administration of rhPDGF-BB alone could not achieve significant periodontal regeneration, especially for patients with severe periodontitis. In this regard, the combinational delivery of two or more bioactive molecules has been proposed to facilitate periodontal regeneration. For example, the combination of PDGF and simvastatin, which acts as an osteogenic differentiation factor, accelerates periodontal regeneration outcomes and leads to increased regeneration of the bone and cementum in addition to promoting the realignment of fibers between these components [41]. However, a recent study on a PDGF-BB gene delivery system has reported that a sustained PDGF-BB production has a negative effect on periodontal regeneration with delayed bone healing [42].

Since PDGFs derived from platelets can enhance periodontal regeneration, PCs containing various growth factors with a certain shape have been extensively studied for the regeneration of periodontal tissues [52]. Currently, PRF, especially PRF-containing leukocytes, which represents the second generation of PCs displaying a certain degree of tensile strength, has been shown to enhance periodontal repair [44,45]. Firstly, PRF contains many crucial angiogenic factors with a strong angiogenic capacity and could further facilitate periodontal regeneration. In addition, PRF that is rich in fibrin and membranes could provide a scaffold for endogenous cells and maintain the spacing in large periodontal defects. Finally, the bioactive factors in PRF could be slowly released and induce long-term activation of the regeneration signaling cascade, resulting in a satisfactory regenerative outcome [46,53]. However, the regeneration induced by PRF depends only on the recruitment of endogenous cells to the defective site. Therefore, its potential is still limited and the majority of regenerated tissues exhibit a disordered structure.

### 3.2. Pharmaceuticals

Although conventional approaches focus on treating the inflammation in periodontal tissues, it is not easy to eliminate inflammation and recreate a healthy microenvironment for periodontal regeneration. In this regard, the application of anti-inflammatory pharmaceuticals is necessary.

For example, aspirin has been used as a non-steroidal anti-inflammatory drug for a long time, with excellent anti-inflammatory properties and limited side effects. The local administration of aspirin in patients with periodontitis could effectively control inflammation and reduce further periodontal impairment. Consequently, the combination of aspirin with PRF and the transplantation of a PRF/aspirin complex into a rat model with periodontal bone defects led to a significant 2-fold improvement in new bone formation compared to the PRF only group [18].

Aside from widely used drugs, some new drugs can also exert anti-inflammatory effects, such as melatonin, which was originally isolated from the bovine pineal tissue [54]. Melatonin controls the circadian rhythm and the sleep-wake cycle. Recently, it was found that the administration of melatonin in a rat periodontitis model increases periodontal regeneration by reducing the levels of inflammatory factors and suppressing oxidative damage [19]. From a mechanistic point of view, melatonin can not only downregulate the level of inflammatory cytokines, such as tumor necrosis factor α (TNF-α), and restore the impaired osteogenic capacity of MSCs [55], but also can alleviate LPS-induced inflammation by blocking nuclear factor κ-light-chain-enhancer of activated B cells (NF-κB) signaling in mice [56].

However, several problems remain to be addressed regarding the use of pharmaceuticals. On the one hand, it is difficult to ensure a controlled release of these compounds in an area with local inflammation while maintaining their sustained potency [57]. On the other hand, long-term administration of pharmaceuticals might lead to drug resistance, which may increase the difficulty of treating patients with periodontitis [58]. Given their side effects, pharmacological approaches have been examined in preclinical studies to inhibit bacterially induced inflammation, facilitate osteogenesis and repair periodontal defects.

### 3.3. Plant Extracts

Natural products, such as herbs used in traditional Chinese medicine and dietary polyphenols extracted from various fruits and plants, are of great importance in the treatment of diseases. Recently, they have received considerable attention as therapeutic options for the treatment of periodontal disease.

One of the most promising drugs for therapeutic application is osthole, a small bioactive molecule extracted from coumarin. Osthole has exhibited diverse pharmacological activities, including anti-inflammatory, anti-oxidative and anti-osteoclastic effects [59]. Pharmacokinetic studies have established that the uptake and utilization of osthole are quick and efficient, indicating that the osthole is effective regardless of the nature of drug administration (orally or locally). Treatment with osthole administered orally in an ovariectomy (OVX) animal model was used to investigate the acute effects of estrogen deficiency. In this study, osthole prevented osteoporosis, increased bone density and suppressed bone loss [60]. Mechanistically, osthole participates in the modulation of bone homeostasis by promoting the proliferation and differentiation of osteoblasts and suppressing the formation and activity of osteoclasts [59]. Additionally, studies in periodontal rat models have determined that a local injection of osthole can also increase periodontal regeneration [47]. Moreover, in vitro studies have indicated that osthole can improve the osteogenic differentiation capacity of PDLSCs via epigenetic modifications, while osthole also induces osteoblastic differentiation by activating the bone morphogenetic proteins signaling pathway and the wingless (Wnt)/β-catenin signaling pathway [59]. In addition, the drug rescues the regenerative capacity of I-PDLSCs, which form the inflammatory microenvironment, and improves bone formation by upregulating the expression of the histone acetylases in I-PDLSCs [17]. These results support the use of osthole as a potential therapeutic agent for enhancing periodontal regeneration.

Another promising agent for periodontal regeneration is resveratrol, one of the dietary flavonoids, which is found in various plants and fruits. This compound has gained attention because of its ideal anti-oxidative and anti-inflammatory capacities, which could be widely used to delay senescence and prevent bone loss [48]. In a rat periodontitis model, the administration of resveratrol orally or locally by injection prevents alveolar bone loss and reduces the levels of inflammatory cytokines and mitochondrial reactive oxygen species (ROS) [20]. Resveratrol promotes the osteogenic potential of endogenous MSCs via the NAD-dependent deacetylase sirtuin-1/forkhead box 3A (SIRT1/FOXO3A) axis and NAD-dependent deacetylase sirtuin-1/5′ adenosine monophosphate-activated protein kinase (SIRT1/AMPK) pathway [48,61]. In vitro studies have shown that treatment with 75 μM resveratrol for 48 h significantly increased the viability of human gingival fibroblasts, with resveratrol not showing any toxicity at concentrations less than 1 mM in MSCs [62].

In addition, quercitrin represents another type of dietary flavonoid, which could inhibit the expression of the cyclooxygenase-2 (COX-2) and prostaglandin E2 (PGE2) proinflammatory cytokines and suppress inflammation-related signaling via the NF-κB pathway to reverse the inflammatory microenvironment [63]. In the OVX rat model, quercitrin was shown to inhibit the progression of osteoporosis and maintain the bone mineral density along with the trabecular microstructure [64]. Moreover, a report on human gingival fibroblasts suggests that quercitrin increases the production of the extracellular matrix (ECM) and downregulates *interleukin-6* (*IL-6*) mRNA and ROS levels, which contribute to the development of periodontitis [49]. Furthermore, quercitrin possesses antibacterial properties, which decrease the bacterial growth rate and thus, directly target the cause of the inflammation. This represents another way of preventing and controlling periodontal disease [50]. However, without direct evidence regarding the application of quercitrin in periodontal defects, it will require a long time before quercitrin becomes a therapeutic agent for periodontal regeneration.

Considering limitations regarding the most suitable concentration and specific mechanisms, only a few studies on periodontal regeneration that focus on these plant extracts exist. However, as the regeneration potential of these plants extracts has been uncovered, they may become promising therapeutic agents in the field of periodontal regeneration.

## 4. Bioactive Molecules Enhance the Effects of Cell Aggregates/Cell Sheets in Periodontal Regeneration

To ensure that the tooth-surrounding region containing the periodontal defect obtains enough support from the periodontal tissues to ensure normal mastication, the aim of periodontal regeneration is to induce the full regeneration of periodontal tissues (including that of the cementum, periodontal ligament and alveolar bone) and to achieve satisfactory reattachment of the tooth. Nowadays, stem cell-based therapies have been extensively developed to improve the outcome of periodontal regeneration based on their dual function of providing enough cells and recreating a favorable microenvironment for regeneration [6]. However, many issues remain unresolved, including choosing the most suitable stem cells, their proper dosage and the best stem cell scaffolds. In this present study, we introduce a new stem cell-based therapy known as cell aggregates/cell sheets technology, which has been successfully used for periodontal regeneration, especially when combined with bioactive molecules that act as signaling molecules.

### 4.1. Cell Aggregates/Cell Sheets as a 3D Scaffolding Material in Periodontal Regeneration

In the field of tissue engineering, scaffolds, which provide the environment and space for stem cells to survive, are important for tissue regeneration. Given that scaffolds provide a foundation for regeneration and will be ultimately applied in humans, they should be safe and highly biocompatible [65]. Studies have shown that exogenous biodegradable scaffolds can induce macrophages and trigger an immune response, which always results in the failure of tissue regeneration because the microenvironment is improper for the survival and differentiation of stem cells [66]. Conversely, transplanted scaffolds should possess the potential to induce the migration and attachment of exogenous and endogenous cells to the defective site, facilitating cellular differentiation and preventing tissue collapse during the initial stages of the regeneration process. To address these problems, the ECM has attracted attention as a scaffold for tissue regeneration due to its ideal properties [67].

The ECM contains various trophic factors, such as collagen, integrins and proteoglycans, and represents a microenvironment that affects the biological characteristics of MSCs. An ECM derived from decellularized tissues generates a favorable microenvironment for resident MSCs and induces the regeneration of the tissue from which the ECM was derived. Moreover, a decellularized ECM derived from PDLSCs provides a tissue-specific microenvironment for PDLSCs, which maintains their stem cell properties, promotes their proliferation and enhances their potential for osteogenic differentiation [37].

As the ECM can determine the fate and function of stem cells in both direct and indirect ways, a new cell delivery approach that employs the ECM as a natural scaffold (known as cell aggregates/cell sheets technology) has been used for periodontal regeneration. Cell sheets were first reported when using temperature-responsive culture dishes, as PDLSCs sheets would form in these culture dishes when the temperature was decreased [68] and lead to the ectopic regeneration of the cementum and of periodontal ligament-like tissues [69]. However, this technology only results in thin cell sheets with little ECM that is insufficient and inconvenient for the regeneration of large periodontal defects.

Therefore, our team has developed a simple but effective approach to generate thick cell sheets for periodontal regeneration, which is also known as cell aggregates. Firstly, PDLSCs were seeded in a normal medium and cultured for 2−3 days. After this, we supplemented the cell culture medium with 50 mg/mL ascorbic acid and continued to culture the cells for 14 days [70]. Finally, we detached the ivory and membrane-like cell aggregates from the edge of dishes. Cell aggregates generated using this strategy secrete high ECM levels and express ECM proteins, such as collagen I, laminin and fibronectin. More importantly, this is not a sensitive technique and is easy to prepare and deliver, leading to effective periodontal regeneration [16,65].

Cell aggregates in combination with ceramic bovine bones (CBBs) [71] or dentin blocks [69] could facilitate the regeneration of complex periodontal structures in immunodeficient mice following ectopic transplantation. Moreover, a randomized clinical trial enlisting 30 patients with periodontitis has reported that the use of autologous PDLSCs aggregates in combination with bone graft materials is safe for treating periodontitis and does not produce significant adverse effects, even though no significant differences were observed when compared with a group without PDLSCs aggregates [33].

Since stem cells that are derived from different tissues possess a higher differentiation potential into the cell types from which they were derived, the use of stem cells derived from different periodontal tissues could result in a better outcome for periodontal regeneration. Consequently, our team has designed a composite cell aggregate system. A treated dentine matrix is used as the tooth without the cementum. After this, two layers of PDLSCs aggregates are applied onto it: one for the regeneration of the cementum and the other for the regeneration of the periodontal ligament. On top of these layers, one layer of bone marrow mesenchymal stem cells (BMMSCs) aggregates, which are MSCs derived from bone marrow, targets the regeneration of the alveolar bone. After transplanting this system ectopically and in situ, the regeneration of cementum-like and periodontal ligament-like tissues was observed, with collagen fibers inserted in both the cementum and the regenerated bone [71]. This study revealed that the co-culture of PDLSCs and BMMSCs aggregates could enhance the crosstalk between these cells and more readily create a favorable regeneration microenvironment, leading to the regeneration of more functional periodontal tissues.

### 4.2. A Combination of Bioactive Molecules and Cell Aggregates/Cell Sheets to Strengthen Periodontal Regeneration

Although the composite system using cell aggregates could enhance the outcome of periodontal regeneration, it is still quite complex for clinical applications. Fortunately, it is widely accepted that signaling molecules could influence the behavior of stem cells by binding to specific receptors on these cells and activating downstream signaling pathways. Therefore, bioactive molecules that play roles similar to those of signaling molecules in directing cell behavior and recreating the cellular microenvironment have been utilized in cell aggregates/cell sheets technology to enhance periodontal regeneration.

It has been reported that the addition of osthole into the culture medium for PDLSCs aggregates could induce the formation of thicker aggregates containing more ECM and trophic factors, such as collagen I and integrin, which are important for cell-to-cell communication and the crosstalk between the cells and the ECM. Furthermore, aggregates pretreated with osthole also exhibit increased differentiation and proliferation [47]. In rat periodontitis models, treatment with a combination of osthole and PDLSCs aggregates significantly enhanced the height of the absorbed alveolar bone and the integration of the periodontal function [17]. In addition to osthole, licochalcone A is another Chinese traditional medicine derived from the root of licorice that can increase ECM expression and the osteogenic differentiation of cell aggregates. Cell aggregates that have been treated with licochalcone A also exhibited higher mineralization potential in healing the bone defects of OVX rats [72]. Further studies have revealed that licochalcone A could enhance the mineralization of cell aggregates by upregulating fatty acid synthase ligand (FasL) and further affecting the extracellular signal-regulated kinases (ERK) pathway [73]. When implanting PDLSCs aggregates in combination with PRP (which is rich in various trophic factors, such as vascular endothelial growth factor and PDGF) subcutaneously into immunocompromised mice, increased bone regeneration is observed compared to that in the control group [74].

Moreover, other bioactive molecules, such as metformin, resveratrol and melatonin, have the ability to enhance the properties of cell aggregates/cell sheets and further improve regeneration outcomes [75]. Among these, our previous study has revealed that melatonin can improve osteogenic differentiation, stemness and the mobility of MSCs, resulting in improved MSCs therapies for bone defects. In a rat calvarial critical-defect regeneration model, a sandwich structure consisting of four layers of melatonin-treated BMMSCs aggregates and three layers of hydroxyapatite/β-tricalcium phosphate (HA/TCP) was delivered. We found that melatonin-treated BMMSC aggregates led to a 3-fold increase in skull restoration when compared with the dimethyl sulfoxide-treated BMMSCs aggregates [55].

However, despite their promising regeneration potential, including a good preservation of cell viability and an abundance of ECM growth factors, there are also drawbacks regarding the use of cell aggregates/cell sheets. Since they are membrane-like structures that lack a certain degree of mechanical strength, they have to be applied together with supporting materials, such as CBBs and HA/TCP. This ensures that the regeneration space is preserved and prevents collapse.

## 5. Conclusions

**Figure 2:**
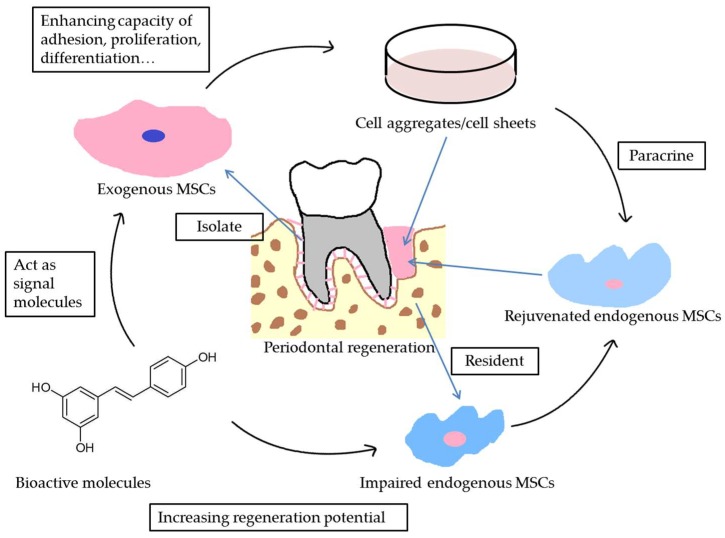
A schematic diagram of the contributions of bioactive molecules in stem cell-based periodontal regeneration. The bioactive molecules could act as signal molecules enhancing the vitality of both endogenous and exogenous MSCs, increasing the regeneration potential of cell aggregates/cell sheets and facilitating periodontal regeneration.

Although cell aggregates/cell sheets containing dynamic MSCs and a rich ECM that favors cellular crosstalk hold great promise for periodontal regeneration, maintaining their therapeutic efficacy in an inflammatory microenvironment remains a major challenge. In this regard, novel bioactive molecules have shown promising effects in reversing impaired microenvironments and modulating cell behavior. Despite limited current evidence regarding their periodontal regenerative capacity, especially in combination with cell aggregates/cell sheets, further investigations in this promising field will be required to confirm their clinical efficacy (Figure 2).

## Figures and Tables

**Table 1:** Summary of the categories of bioactive molecules that are involved in periodontal regeneration.

Category	Bioactive Molecules	Effect	Experimental Model	Studies
Growth factors	EMD	Facilitate osteoblasts and PDLSCs adhesion, proliferation	Osteoblasts	[34,35,36]
			PDLSCs	[34,37]
		Promote periodontal regeneration	Class III furcation defects in monkeys	[38]
			Human with periodontitis	[39,40]
	PDGF	Accelerates the regeneration of the periodontal apparatus	Periodontal defect in rat	[41,42]
		Periodontal ligament interposed between newly formed cementum and alveolar bone	Human with class II furcation lesions	[43]
	PRP/PRF	Strong angiogenic capacity	Human with periodontitis	[44,45,46]
		Provide a nature scaffold		
		Slowly release bioactive factors		
Pharmaceuticals	Aspirin	Control inflammation	Periodontal defect in rat	[18]
	Melatonin	Anti-inflammation and anti-oxidative	Experimental periodontitis in rat	[19]
Plant extracts	Osthole	Improve the capacity of osteogenic differentiation	PDLSCs	[17,47]
		Promote periodontal regeneration	Periodontal defect in rat	[17]
	Resveratrol	Prevent bone loss and promote osteogenesis	Periodontitis model in rat	[20,48]
	Quercitrin	Anti-inflammation	Human gingival fibroblasts	[49,50]

EMD—Enamel matrix derivative; PDGF—Platelet-derived growth factor; PDLSCs—Periodontal ligament stem cells; PRP—Platelet-rich plasma; PRF—Platelet-rich fibrin.

## Abbreviations

MSCs	Mesenchymal stem cells
PRP	Platelet-rich plasma
PRF	Platelet-rich fibrin
LPS	Lipopolysaccharide
PDLSCs	Periodontal ligament stem cells
I-PDLSCs	Inflamed periodontal ligament stem cells
H-PDLSCs	Healthy periodontal ligament stem cells
OVX	Ovariectomy
BMMSCs	Bone marrow mesenchymal stem cells
ECM	Extracellular matrix
EMD	Enamel matrix derivative
PDGF	Platelet-derived growth factor

## References

[1] Armitage G.C. (1999). Development of a classification system for periodontal diseases and conditions. Ann. Periodontol..

[2] Pihlstrom B., Michalowicz B., Johnson N. (2005). Periodontal diseases. Lancet.

[3] Amano A. (2017). Periodontal diseases and systemic diseases. Clin. Calcium.

[4] Paster B.J., Boches S.K., Galvin J.L., Ericson R.E., Lau C.N., Levanos V.A., Sahasrabudhe A., Dewhirst F.E. (2001). Bacterial diversity in human subgingival plaque. J. Bacteriol..

[5] Nugala B., Kumar B.S., Sahitya S., Krishna P.M. (2012). Biologic width and its importance in periodontal and restorative dentistry. J. Conserv. Dent..

[6] Chen F.M., Sun H.H., Lu H., Yu Q. (2012). Stem cell-delivery therapeutics for periodontal tissue regeneration. Biomaterials.

[7] Slavin S., Nagler A., Naparstek E., Kapelushnik Y., Aker M., Cividalli G., Varadi G., Kirschbaum M., Ackerstein A., Samuel S. (1998). Nonmyeloablative stem cell transplantation and cell therapy as an alternative to conventional bone marrow transplantation with lethal cytoreduction for the treatment of malignant and nonmalignant hematologic diseases. Blood.

[8] Rohban R., Pieber T.R. (2017). Mesenchymal Stem and Progenitor Cells in Regeneration, Tissue Specificity and Regenerative Potential. Stem Cells Int..

[9] Seo B.M., Miura M., Gronthos S., Bartold P.M., Batouli S., Brahim J., Young M., Robey P.G., Wang C.Y., Shi S. (2004). Investigation of multipotent postnatal stem cells from human periodontal ligament. Lancet.

[10] Serra T., Planell J.A., Navarro M. (2013). High-resolution PLA-based composite scaffolds via 3-D printing technology. Acta Biomater..

[11] Huebsch N., Lippens E., Lee K., Mehta M., Koshy S.T., Darnell M.C., Desai R.M., Madl C.M., Xu M., Zhao X. (2015). Matrix elasticity of void-forming hydrogels controls transplanted-stem-cell-mediated bone formation. Nat. Mater..

[12] Wang J., Yang M., Zhu Y., Wang L., Tomsia A.P., Mao C. (2014). Phage nanofibers induce vascularized osteogenesis in 3D printed bone scaffolds. Adv. Mater..

[13] Subramony S.D., Dargis B.R., Castillo M., Azeloglu E.U., Tracey M.S., Su A., Lu H.H. (2013). The guidance of stem cell differentiation by substrate alignment and mechanical stimulation. Biomaterials.

[14] Hu X., Wang Y., Tan Y., Wang J., Liu H., Wang Y., Yang S., Shi M., Zhao S., Zhang Y. (2017). A Difunctional Regeneration Scaffold for Knee Repair based on Aptamer-Directed Cell Recruitment. Adv. Mater..

[15] Bez M., Sheyn D., Tawackoli W., Avalos P., Shapiro G., Giaconi J.C., Da X., David S.B., Gavrity J., Awad H.A. (2017). In situ bone tissue engineering via ultrasound-mediated gene delivery to endogenous progenitor cells in mini-pigs. Sci. Transl. Med..

[16] Yang Z., Jin F., Zhang X., Liu X., Zhang Y., Liu J., Duan Y., Jin Y. (2010). A Novel Possible Strategy Based on Self-Assembly Approach to Achieve Complete Periodontal Regeneration. Artif. Organs.

[17] Sun J., Dong Z., Zhang Y., He X., Fei D., Jin F., Yuan L., Li B., Jin Y. (2017). Osthole improves function of periodontitis periodontal ligament stem cells via epigenetic modification in cell sheets engineering. Sci. Rep..

[18] Du J., Mei S., Guo L., Su Y., Wang H., Liu Y., Zhao Z., Wang S., Liu Y. (2018). Platelet-rich fibrin/aspirin complex promotes alveolar bone regeneration in periodontal defect in rats. J. Periodontal Res..

[19] Kara A., Akman S., Ozkanlar S., Tozoglu U., Kalkan Y., Canakci C.F., Tozoglu S. (2013). Immune modulatory and antioxidant effects of melatonin in experimental periodontitis in rats. Free Radic. Biol. Med..

[20] Bhattarai G., Poudel S.B., Kook S., Lee J. (2016). Resveratrol prevents alveolar bone loss in an experimental rat model of periodontitis. Acta Biomater..

[21] Tang H., Xia Y., Yu Y., Wu R., Gao L., Chen F. (2016). Stem cells derived from “inflamed” and healthy periodontal ligament tissues and their sheet functionalities, a patient-matched comparison. J. Clin. Periodontol..

[22] Sui B.D., Hu C.H., Liu A.Q., Zheng C.X., Xuan K., Jin Y. (2017). Stem cell-based bone regeneration in diseased microenvironments, Challenges and solutions. Biomaterials.

[23] Liu D., Xu J., Liu O., Fan Z., Liu Y., Wang F., Ding G., Wei F., Zhang C., Wang S. (2012). Mesenchymal stem cells derived from inflamed periodontal ligaments exhibit impaired immunomodulation. J. Clin. Periodontol..

[24] Liu J., Wang L., Liu W., Li Q., Jin Z., Jin Y. (2014). Dental follicle cells rescue the regenerative capacity of periodontal ligament stem cells in an inflammatory microenvironment. PLoS ONE.

[25] Zheng W., Wang S., Ma D., Tang L., Duan Y., Jin Y. (2009). Loss of proliferation and differentiation capacity of aged human periodontal ligament stem cells and rejuvenation by exposure to the young extrinsic environment. Tissue Eng..

[26] Li N., Liu N., Zhou J., Tang L., Ding B., Duan Y., Jin Y. (2013). Inflammatory environment induces gingival tissue-specific mesenchymal stem cells to differentiate towards a pro-fibrotic phenotype. Biol. Cell.

[27] Chen F., Liu X. (2016). Advancing biomaterials of human origin for tissue engineering. Prog. Polym. Sci..

[28] Kusuma G.D., Carthew J., Lim R., Frith J.E. (2017). Effect of the Microenvironment on Mesenchymal Stem Cell Paracrine Signaling, Opportunities to Engineer the Therapeutic Effect. Stem Cells Dev..

[29] Phinney D.G., Pittenger M.F. (2017). Concise Review, MSC-Derived Exosomes for Cell-Free Therapy. Stem Cells.

[30] Rajan T.S., Giacoppo S., Trubiani O., Diomede F., Piattelli A., Bramanti P., Mazzon E. (2016). Conditioned medium of periodontal ligament mesenchymal stem cells exert anti-inflammatory effects in lipopolysaccharide-activated mouse motoneurons. Exp. Cell Res..

[31] Ohyashiki J.H., Umezu T., Ohyashiki K. (2018). Extracellular vesicle-mediated cell-cell communication in haematological neoplasms. Philos. Trans. R. Soc. Lond. B Biol. Sci..

[32] Panduwawala C.P., Zhan X., Dissanayaka W.L., Samaranayake L.P., Jin L., Zhang C. (2017). In vivo periodontal tissue regeneration by periodontal ligament stem cells and endothelial cells in three-dimensional cell sheet constructs. J. Periodontal Res..

[33] Chen F.M., Gao L.N., Tian B.M., Zhang X.Y., Zhang Y.J., Dong G.Y., Lu H., Chu Q., Xu J., Yu Y. (2016). Treatment of periodontal intrabony defects using autologous periodontal ligament stem cells: A randomized clinical trial. Stem Cell Res. Ther..

[34] Miron R.J., Chandad F., Buser D., Sculean A., Cochran D.L., Zhang Y. (2016). Effect of Enamel Matrix Derivative Liquid on Osteoblast and Periodontal Ligament Cell Proliferation and Differentiation. J. Periodontol..

[35] Miron R.J., Fujioka-Kobayashi M., Zhang Y., Caballe-Serrano J., Shirakata Y., Bosshardt D.D., Buser D., Sculean A. (2017). Osteogain improves osteoblast adhesion, proliferation and differentiation on a bovine-derived natural bone mineral. Clin. Oral Implants Res..

[36] Miron R.J., Sculean A., Cochran D.L., Froum S., Zucchelli G., Nemcovsky C., Donos N., Lyngstadaas S.P., Deschner J., Dard M. (2016). Twenty years of enamel matrix derivative, the past, the present and the future. J. Clin. Periodontol..

[37] Zhang J., Song Z., Xia Y., Shu R. (2017). Extracellular matrix derived from periodontal ligament cells maintains their stemness and enhances redifferentiation via the wnt pathway. J. Biomed. Mater. Res. Part A.

[38] Shirakata Y., Miron R.J., Nakamura T., Sena K., Shinohara Y., Horai N., Bosshardt D.D., Noguchi K., Sculean A. (2017). Effects of EMD liquid (Osteogain) on periodontal healing in class III furcation defects in monkeys. J. Clin. Periodontol..

[39] Gupta S.J., Jhingran R., Gupta V., Bains V.K., Madan R., Rizvi I. (2014). Efficacy of platelet-rich fibrin vs. enamel matrix derivative in the treatment of periodontal intrabony defects, a clinical and cone beam computed tomography study. J. Int. Acad. Periodontol..

[40] Aimetti M., Ferrarotti F., Mariani G., Fratini A., Giraudi M., Romano F. (2016). Enamel Matrix Derivative Proteins in Combination with a Flapless Approach for Periodontal Regeneration of Intrabony Defects, A 2-Year Prospective Case Series. Int. J. Periodontics Restor. Dent..

[41] Chang P., Dovban A.S., Lim L.P., Chong L.Y., Kuo M.Y., Wang C. (2013). Dual delivery of PDGF and simvastatin to accelerate periodontal regeneration in vivo. Biomaterials.

[42] Plonka A.B., Khorsand B., Yu N., Sugai J.V., Salem A.K., Giannobile W.V., Elangovan S. (2016). Effect of sustained PDGF nonviral gene delivery on repair of tooth-supporting bone defects. Gene Ther..

[43] Kaigler D., Avila G., Wisner-Lynch L., Nevins M.L., Nevins M., Rasperini G., Lynch S.E., Giannobile W.V. (2011). Platelet-derived growth factor applications in periodontal and peri-implant bone regeneration. Expert Opin. Biol. Ther..

[44] Castro A.B., Meschi N., Temmerman A., Pinto N., Lambrechts P., Teughels W., Quirynen M. (2017). Regenerative potential of leucocyte- and platelet-rich fibrin. Part, A.; intra-bony defects, furcation defects and periodontal plastic surgery. A systematic review and meta-analysis. J. Clin. Periodontol..

[45] Aydemir Turkal H., Demirer S., Dolgun A., Keceli H.G. (2016). Evaluation of the adjunctive effect of platelet-rich fibrin to enamel matrix derivative in the treatment of intrabony defects. Six-month results of a randomized, split-mouth, controlled clinical study. J. Clin. Periodontol..

[46] Baba S., Yamada Y., Komuro A., Yotsui Y., Umeda M., Shimuzutani K., Nakamura S. (2016). Phase I/II Trial of Autologous Bone Marrow Stem Cell Transplantation with a Three-Dimensional Woven-Fabric Scaffold for Periodontitis. Stem Cells Int..

[47] Gao L., An Y., Lei M., Li B., Yang H., Lu H., Chen F., Jin Y. (2013). The effect of the coumarin-like derivative osthole on the osteogenic properties of human periodontal ligament and jaw bone marrow mesenchymal stem cell sheets. Biomaterials.

[48] Tamaki N., Cristina O.R., Inagaki Y., Fukui M., Nagata T., Ito H.O. (2014). Resveratrol improves oxidative stress and prevents the progression of periodontitis via the activation of the Sirt1/AMPK and the Nrf2/antioxidant defense pathways in a rat periodontitis model. Free Radic. Biol. Med..

[49] Gómez-Florit M., Monjo M., Ramis J.M. (2015). Quercitrin for periodontal regeneration, effects on human gingival fibroblasts and mesenchymal stem cells. Sci. Rep..

[50] Gomez-Florit M., Monjo M., Ramis J.M. (2014). Identification of quercitrin as a potential therapeutic agent for periodontal applications. J. Periodontol..

[51] Chen W., Baylink D.J., Brier-Jones J., Neises A., Kiroyan J.B., Rundle C.H., Lau K.H., Zhang X.B. (2015). PDGFB-based stem cell gene therapy increases bone strength in the mouse. Proc. Natl. Acad. Sci. USA.

[52] Wang Z., Feng Z., Wu G., Bai S., Dong Y., Chen F., Zhao Y. (2016). The use of platelet-rich fibrin combined with periodontal ligament and jaw bone mesenchymal stem cell sheets for periodontal tissue engineering. Sci. Rep..

[53] Nakajima D., Tabata Y., Sato S. (2015). Periodontal tissue regeneration with PRP incorporated gelatin hydrogel sponges. Biomed. Mater..

[54] Jahanban-Esfahlan R., Mehrzadi S., Reiter R.J., Seidi K., Majidinia M., Baghi H.B., Khatami N., Yousefi B., Sadeghpour A. (2017). Melatonin in regulation of inflammatory pathways in rheumatoid arthritis and osteoarthritis, involvement of circadian clock genes. Br. J. Pharmacol..

[55] Shuai Y., Liao L., Su X., Yu Y., Shao B., Jing H., Zhang X., Deng Z., Jin Y. (2016). Melatonin Treatment Improves Mesenchymal Stem Cells Therapy by Preserving Stemness during Long-term In Vitro Expansion. Theranostics.

[56] Carpentieri A.R., Peralta Lopez M.E., Aguilar J., Solá V.M. (2017). Melatonin and periodontal tissues, Molecular and clinical perspectives. Pharmacol. Res..

[57] Sundararaj S.C., Thomas M.V., Peyyala R., Dziubla T.D., Puleo D.A. (2013). Design of a multiple drug delivery system directed at periodontitis. Biomaterials.

[58] Olsen I. (2015). Biofilm-specific antibiotic tolerance and resistance. Eur. J. Clin. Microbiol. Infect. Dis..

[59] Zhang Z., Leung W.N., Cheung H.Y., Chan C.W. (2015). Osthole, A Review on Its Bioactivities, Pharmacological Properties, and Potential as Alternative Medicine. Evid.-Based Complement. Altern. Med..

[60] Zhang Z., Leung W.N., Li G., Lai Y.M., Chan C.W. (2016). Osthole Promotes Endochondral Ossification and Accelerates Fracture Healing in Mice. Calcif. Tissue Int..

[61] Tseng P.C., Hou S.M., Chen R.J., Peng H.W., Hsieh C.F., Kuo M.L., Yen M.L. (2011). Resveratrol promotes osteogenesis of human mesenchymal stem cells by upregulating RUNX2 gene expression via the SIRT1/FOXO3A axis. J. Bone Miner. Res..

[62] Pangeni R., Sahni J.K., Ali J., Sharma S., Baboota S. (2014). Resveratrol, review on therapeutic potential and recent advances in drug delivery. Expert Opin. Drug Deliv..

[63] Comalada M., Camuesco D., Sierra S., Ballester I., Xaus J., Galvez J., Zarzuelo A. (2005). In vivo quercitrin anti-inflammatory effect involves release of quercetin, which inhibits inflammation through down-regulation of the NF-kappaB pathway. Eur. J. Immunol..

[64] Xing L., Ni H., Wang Y. (2017). Quercitrin attenuates osteoporosis in ovariectomized rats by regulating mitogen-activated protein kinase (MAPK) signaling pathways. Biomed. Pharmacother..

[65] Yang J., Yamato M., Kohno C., Nishimoto A., Sekine H., Fukai F., Okano T. (2005). Cell sheet engineering, recreating tissues without biodegradable scaffolds. Biomaterials.

[66] Moioli E.K., Clark P.A., Xin X., Lal S., Mao J.J. (2007). Matrices and scaffolds for drug delivery in dental, oral and craniofacial tissue engineering. Adv. Drug Deliv. Rev..

[67] Gattazzo F., Urciuolo A., Bonaldo P. (2014). Extracellular matrix: A dynamic microenvironment for stem cell niche. Biochim. Biophys. Acta (BBA) Gen. Subj..

[68] Kushida A., Yamato M., Konno C., Kikuchi A., Sakurai Y., Okano T. (1999). Decrease in culture temperature releases monolayer endothelial cell sheets together with deposited fibronectin matrix from temperature-responsive culture surfaces. J. Biomed. Mater. Res..

[69] Washio K., Iwata T., Mizutani M., Ando T., Yamato M., Okano T., Ishikawa I. (2010). Assessment of cell sheets derived from human periodontal ligament cells, a pre-clinical study. Cell Tissue Res..

[70] Yang Z., Jin F., Zhang X., Ma D., Han C., Huo N., Wang Y., Zhang Y., Lin Z., Jin Y. (2009). Tissue engineering of cementum/periodontal-ligament complex using a novel three-dimensional pellet cultivation system for human periodontal ligament stem cells. Tissue Eng. Part C Methods.

[71] Zhang H., Liu S., Zhu B., Xu Q., Ding Y., Jin Y. (2016). Composite cell sheet for periodontal regeneration, crosstalk between different types of MSCs in cell sheet facilitates complex periodontal-like tissue regeneration. Stem Cell Res. Ther..

[72] Shang F., Ming L., Zhou Z., Yu Y., Sun J., Ding Y., Jin Y. (2014). The effect of licochalcone A on cell-aggregates ECM secretion and osteogenic differentiation during bone formation in metaphyseal defects in ovariectomized rats. Biomaterials.

[73] Ming L., Jin F., Huang P., Luo H., Liu W., Zhang L., Yuan W., Zhang Y., Jin Y. (2015). Licochalcone A up-regulates of FasL in mesenchymal stem cells to strengthen bone formation and increase bone mass. Sci. Rep..

[74] Xu Q., Li B., Yuan L., Dong Z., Zhang H., Wang H., Sun J., Ge S., Jin Y. (2017). Combination of platelet-rich plasma within periodontal ligament stem cell sheets enhances cell differentiation and matrix production. J. Tissue Eng. Regen. Med..

[75] Zhao P., Sui B.D., Liu N., Lv Y.J., Zheng C.X., Lu Y.B., Huang W.T., Zhou C.H., Chen J., Pang D.L. (2017). Anti-aging pharmacology in cutaneous wound healing effects of metformin, resveratrol, and rapamycin by local application. Aging Cell.

